# The efficacy and safety of Xiangsha Liujunzi decoction in the treatment of chronic non-atrophic gastritis: A protocol for a systematic review and meta-analysis

**DOI:** 10.1097/MD.0000000000024504

**Published:** 2021-01-29

**Authors:** Pan Yue, Juan Zhong, Jiajun Huang, Zhaoxi Lan, Sen Zhong

**Affiliations:** a Hospital of Chengdu University of Traditional Chinese Medicine; b School of Clinical Medicine, Chengdu University of Traditional Chinese Medicine; c Affiliated Sport Hospital of Chengdu Sport University, Chengdu, Sichuan Province, P.R. China.

**Keywords:** chronic non-atrophic gastritis, complementary and alternative therapy, protocol, systematic review, Xiangsha Liujunzi decoction

## Abstract

**Background:**

Chronic non-atrophic gastritis is the most common type of chronic gastritis. Even if treated with current standard pharmacotherapy, the efficacy is not very satisfactory due to many factors. Traditional Chinese Medicine decoctions are increasingly used in the treatment of chronic gastritis, and many studies have shown that Xiangsha Liujunzi decoction is effective and safe in the treatment of chronic non-atrophic gastritis. However, it is controversy if Xiangsha Liujunzi decoction can provide an evidence-based clinical efficacy and safety in the treatment of chronic non-atrophic gastritis.

**Methods:**

We will go through 8 databases, and conduct a systematic review of Xiangsha Liujunzi decoction and health-related outcomes in chronic non-atrophic gastritis patients according to the Preferred Reporting Items for Systematic Reviews. The primary objective is to assess the impact of Preferred Reporting Items for Systematic Reviews and Meta-Analyses guidelines on clinical outcomes relevant to chronic non-atrophic gastritis patients, such as clinical effective rate,

*Helicobacter pylori* eradication rate, efficacy under endoscopy, 1-year recurrent rate, and number of reported adverse events. Cochrane Risk Assessment Tool will be used to assess the quality of eligible studies according to the Cochrane handbook.

**Results:**

The results of this systematic review will provide a synthesis of current evidence of Xiangsha Liujunzi decoction and we have a specific opportunity to determine the efficacy and safety of it.

**Conclusion:**

This study will explore whether or not Xiangsha Liujunzi decoction can be used as one of the complementary and alternative therapies in the treatment of chronic non-atrophic gastritis.

**Registration number:**

Identifier: DOI 10.17605/OSF.IO/TX27U (https://osf.io/tx27u/).

## Abstract 2

Supplemental Digital Content is available in the text

## 1 Introduction

### 1.1 Description of the condition

Chronic gastritis (CG) is a very common disease of the digestive system. It is a chronic inflammatory condition of the gastric mucosa induced by various causes, mainly by *Helicobacter pylori* (HP) infection.^[1]^ CG can be categorized into chronic non-atrophic gastritis (CNG), chronic atrophic gastritis (CAG), and special types of gastritis.^[2]^ Although its prevalence rate in the general population still remains unknown, a national multi-center cross-sectional study demonstrated that 49.3% of the 8892 investigated patients with upper gastrointestinal symptoms who underwent diagnostic upper endoscopy from 33 centers had CNG, which was the most common type of CG.^[3]^ Without proper treatment, CNG may develop into CAG, which is an important precursor lesion in the development of gastric carcinoma.^[4]^

### 1.2 Description of the intervention

Routine treatment of CNG generally includes eliminating the causes of illness such as nonsteroidal anti-inflammatory drug use, diet control, and pharmacotherapy for the eradication of HP and amelioration of the symptoms.^[1]^ Medications for CNG mainly include antibiotics, proton pump inhibitors (PPIs), gastric mucosal protective agents, antiacids, and prokinetics.^[5]^ However, even if treated with standard medications, the efficacy is less than satisfactory and some adverse effects may occur.^[6]^ For instance, recent studies have shown that long-term PPI use may change the colonization mode of HP and this could accelerate the process of gland loss and subsequently result in the appearance of CAG.^[7]^ Besides, the eradication rate of HP has decreased due to the increase of antimicrobial resistance, poor compliance, and adverse effects.^[8]^ Therefore, it is necessary to turn to complementary and alternative medicine for more effective and less harmful therapies. Based on its clinical manifestations, CNG can be roughly categorized as Weiwantong (stomachache), Piman (abdominal distention), or Caoza (stomach upset and discomfort).^[9]^ The results of a recent meta-analysis on the efficacy of traditional Chinese medicine (TCM) for CG indicate that a variety of TCM decoctions including Banxia Xiexin decoction, Shenyang Yiwei decoction, Xiangsha Liujunzi decoction, and other classic formulae could offer certain advantages in the treatment of CG, especially in the ease of the above symptoms.^[10]^

### 1.3 How the intervention might work

First composed by Yunbo Ke, a TCM physician in the Qing dynasty, Xiangsha Liujunzi decoction (XSLJZD) has long been used to treat gastrointestinal discomfort caused by gastritis in the clinical practice since the 17th century.^[11,12]^ It consists of 8 different herbs: Aucklandia lappa, Amomum uillosum, Citrus reticulata, Pinellia ternata, Panax ginseng, Atractylodes macrocephala, Porida cocos, and Glycyrrhiza uralensis, each has its own distinctive effect on the digestive system as a Chinese medicinal herb. According to the TCM theory, this formula could invigorate the spleen to resolve dampness, regulate the stomach, facilitate the digestive tract elimination, and replenish Qi (body energy).^[13]^ Studies have shown that XSLJZD could eradicate HP to alleviate CAG mucosal inflammation,^[14]^ regulate gastrointestinal motility,^[15]^ increase plasma motilin secretion,^[16]^ lower serum gastrin levels, and enhance smooth muscle contraction by increasing calcium levels.^[17]^

### 1.4 Why it is important to do this systematic review and meta-analysis

There are randomized controlled trials (RCTs) published in China indicating that XSLJZD as a complementary therapy combined with conventional treatment could better improve the clinical cure rate, HP infection clearance rate, and efficacy under endoscopy of CNG.^[18,19]^ However, there is no critically appraised evidence as systematic review or meta-analysis of the efficacy and safety of XSLJZD for CNG. If XSLJZD proved to be truly effective and safe, it could be recognized and applied by more physicians as a complementary therapy in the treatment of CNG, and benefit more patients.

### 1.5 Objectives

In this study we intend to systematically review published RCTs about XSLJZD in the treatment of CNG and conduct a meta-analysis about its efficacy and safety. We hope this study could provide reliable evidence as reference basis for the clinical application of this classic TCM formula as well as clinical guidelines.

## 2 Methods

This study had been registered in https://osf.io/tx27u/. The registration number is: Identifier: DOI 10.17605/OSF.IO/TX27U

This meta-analysis will be based on the preferred reporting items for the systematic review and meta-analysis (PRISMA) of the project.^[20]^

### 2.1 Inclusion criteria for study selection

#### 2.1.1 Type of studies

All the RCTs to explore the efficacy and safety of XSLJZD in the treatment of CNG will be included. Cross-trials, quasi-RCTs, case reports, observation studies, animal studies, repeatedly published studies, and studies did not have access to complete data will be excluded. Language and time of publication will not be restricted. If we are unable to find at least 5 eligible RCTs for the systematic review, we will broaden our inclusion criteria to include semi-randomized control studies, nonrandomized studies of XSLJZD in the treatment of CNG using the Cochrane Effective Practice and Organization of Care approach to categorize the types of studies.^[21]^

#### 2.1.2 Types of participants

Participants who meet the diagnostic criteria of CNG will be included, regardless of their gender, age, and race. However, CAG or CNG combined with gastric ulcer, duodenal ulcer or other orthopedic diseases were excluded. Diagnostic criteria will be based on the consensus on CG in China (Shanghai 2012).^[5]^

#### 2.1.3 Types of interventions and controls

Interventions include XSLJZD with other conventional therapies in the observational group, and there are no restrictions on the dosage of each herb of this formula as long as within their upper limit per day. In the control group interventions only include conventional treatment. All the conventional therapies are based on the consensus on CG in China (Shanghai 2012).^[5]^

#### 2.1.4 Types of outcome measures

The primary outcomes are the clinical effective rate and HP eradication rate. The secondary outcomes include efficacy under endoscopy, 1-year recurrent rate, and number of reported adverse events associated with the use of XSLJZD.

### 2.2 Search methods for identification of studies

#### 2.2.1 Data sources

PubMed, EMBASE (Excerpta Medical Database), the Cochrane Library, the Chinese Cochrane Centre's Controlled Trials Register platform, the Wanfang Chinese digital periodical and conference database, China National Knowledge Infrastructure database, and the VIP Chinese Science and Technique Journals Database will be searched by our authors for relevant literature. The data will be searched in English and Chinese databases from their inception to December, 2020.

#### 2.2.2 Other search resources

Chinese Clinical Trial Registry Center will also be screened for ongoing trials. We will also review the references of included manuscripts to identify any information about missed trials. We will contact the author if we cannot clearly identify information from the data.

#### 2.2.3 Search strategy

We will employ a broad electronic search strategy in Supplemental Digital Content (Appendix A, http://links.lww.com/MD/F605).

### 2.3 Data extraction, quality, and validation

#### 2.3.1 Study selection and inclusion

Researchers will import the literature retrieved to the Endnote X7 and eliminate the duplicate data. All titles and abstracts returned using the search strategy above will be screened by 2 independent investigators (JZ, ZXL) in line with our advanced inclusion criteria. And then, the full text of the entire study will be reviewed by 3 authors for analysis. Any differences will be resolved by consensus. Finally, another study member will resolve the inconsistencies and check the final literature that will be included.

#### 2.3.2 Data extraction and management

**Figure 1:**
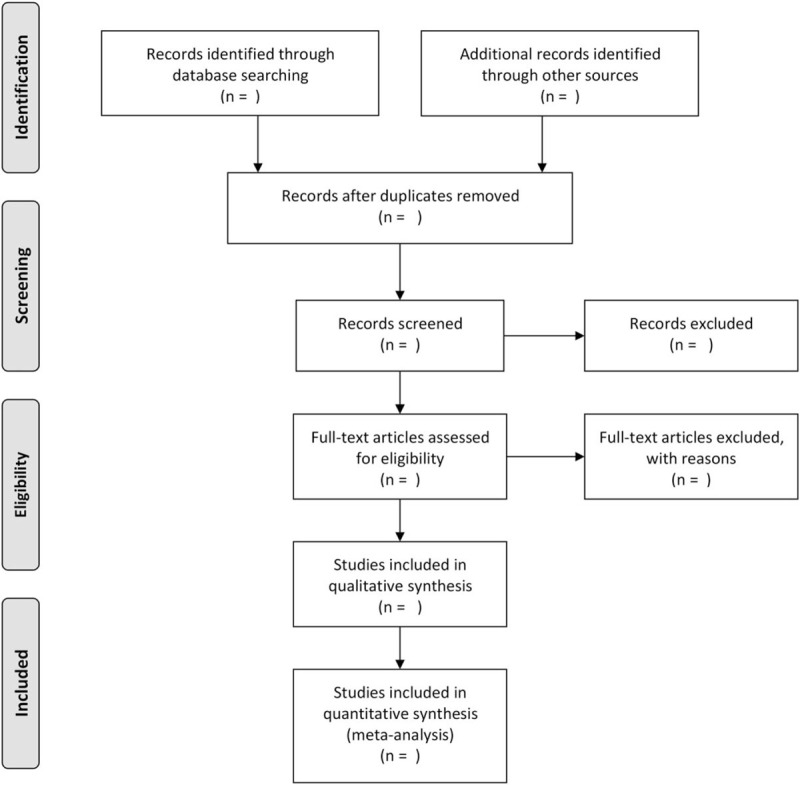
Flow diagram of study selection process.

The raw data from the papers will be extracted by 3 authors (PY, SZ, JJH) and will include: author details, publication information, sample size, and original study design information, such as intervention and comparison (dose, route, and time), outcome measures, and follow-up information. Catgut brand information will be also extracted from us if possible. All extracted data will be verified by a second investigator to ensure accuracy and completeness. All outcome variables will be collected, regardless of the number of studies that the outcome assessed. If conflict, arbitration will be conducted through discussion or through the third reviewer (JJH). PRISMAdiagram (Fig. 1) based on the search strategy and eligibility assessment to show the flow of included and excluded studies will be developed by us.

### 2.4 Assessment of risk of bias

The methodological quality of the included RCTs will be assessed based on the instrument developed in the Cochrane Handbook for Systematic of Interventions by 3 investigators. The tool evaluates studies based on 7 criteria:

(1) randomization generation,
(2) allocation concealment,
(3) blinding of outcome assessors,
(4) blinding patients/study personnel,
(5) incomplete outcome data (that is, lost to follow-up),
(6) selective outcome reporting, and
(7) other risks of bias.

We will define other bias as trials which may be sponsored by XSLJZD manufacturers, and in which baseline characteristics are not similar between the different intervention groups. We will also assess publication bias by examining funnel plots if there are 10 or more trials reporting the primary outcomes.

### 2.5 Quantitative data and statistical methods

#### 2.5.1 Quantitative data synthesis

Review Manager (RevMan) software version 5.3 will be applied to pool our data to perform the meta-analysis. Measurements of dichotomous data will to be expressed as relative risks along with 95% confidence intervals (CIs); for continuous data, mean difference, 95% CIs will be adopted, and *P* < .05 will be defined as statistically significant.

#### 2.5.2 Assessment of heterogeneity

In our review, *I*^2^ values will be used to assess inter-study heterogeneity. When *I*^2^ > 75%, considerable heterogeneity will be conformed, whereupon a random effects model will be applied. We will pool trials when the intervention form of those studies is adequately similar. Specific subgroups will be analyzed according to similar intervention forms or similar design.

#### 2.5.3 Assessment of reporting bias

If a sufficient number of studies are available (at least 10 studies), we will attempt to assess publication bias using a funnel plot.

#### 2.5.4 Subgroup analysis and investigation of heterogeneity

If there is a significant heterogeneity in the included trials, we will conduct subgroup analysis based on the type of disease, differences in treatment frequencies and follow-up duration will also be included.

#### 2.5.5 Sensitivity analysis

If the test for heterogeneity *P*-value is less than .1 after performing the subgroup analysis, the sensitivity analysis will be conducted to evaluate the robustness of our results. The meta-analysis will be repeated after omitting the low-quality studies. Moreover, we will also assess whether the statistics model (random-effects model and fixed-effects model) will affect the current results.

#### 2.5.6 Grading the quality of evidence

We will apply the Grading of Recommendation Assessment, Development, and Evaluation method to evaluate the level of confidence in regards to outcomes. Two independent reviewers will conduct the assessment. In most cases, disagreements were resolved by discussion. If disagreement remained after discussion, a third reviewer will be consulted before taking the final decision on the disagreement.

## 3 Discussion

CNG was the most common type of CG. Without effective treatment, patients may suffer from upper gastrointestinal symptoms, influencing their normal life and work, and it may develop into CAG, a precursor lesion of gastric carcinoma.^[22]^ The efficacy of standard pharmacotherapy is not very satisfactory due to many factors such as the increase of antimicrobial resistance of HP, poor patient compliance or adverse effects of PPIs. XSLJZD is a famous effective TCM formula in the treatment of CNG related symptoms for hundreds of years, and a series of clinical and experimental studies have demonstrated the efficacy and potential mechanisms of this formula. In this study, we will conduct a systematic review and meta-analysis to help determine the efficacy and safety of XSLJZD in the treatment of CNG. The findings may provide sound evidence as reference basis for clinical guidelines and for clinicians to utilize XSLJZD as a complementary and alternative therapy in the treatment of CNG and may also trigger further researches on the mechanisms of its curative effects.

## Author contributions

**Conceptualization:** Pan Yue.

**Data curation:** Pan Yue, Zhaoxi Lan.

**Formal analysis:** Zhaoxi Lan.

**Funding acquisition:** Sen Zhong.

**Investigation:** Pan Yue.

**Methodology:** Juan Zhong.

**Project administration:** Pan Yue.

**Resources:** Jiajun Huang.

**Software:** Juan Zhong.

**Supervision:** Zhaoxi Lan, Sen Zhong.

**Visualization:** Jiajun Huang, Sen Zhong.

**Writing – original draft:** Pan Yue, Juan Zhong.

**Writing – review & editing:** Jiajun Huang.

## Abbreviations

Abbreviations: 95%CI = 95% confidence interval, CNG = chronic non-atrophic gastritis, HP = *Helicobacter pylori*, RCTs = randomized controlled trials, RR = relative risk, TCM = traditional Chinese medicine, XSLJZD = Xiangsha Liujunzi decoction.
